# Development and Evaluation of a Training Program for Community-Based Participatory Research in Breast Cancer

**DOI:** 10.3390/ijerph16224310

**Published:** 2019-11-06

**Authors:** Marj Plumb, Senaida Fernandez Poole, Heather Sarantis, Susan Braun, Janna Cordeiro, Juliana Van Olphen, Marion Kavanaugh-Lynch

**Affiliations:** 1 Plumbline Coaching and Consulting, Omaha, NE 68106, USA; marjplumb@me.com; 2 California Breast Cancer Research Program, University of California Office of the President, Oakland, CA 94612, USA; Senaida.poole@ucop.edu; 3 Independent Researcher, Berkeley, CA 94703, USA; heather@commonweal.org; 4 The V Foundation for Cancer Research, Cary, NC 27513, USA; sbraun@v.org; 5 Cordeiro Consulting, San Francisco, CA 94107, USA; janna.cordeiro@mac.com; 6 Health Education Department, College of Health and Social Sciences, San Francisco State University, San Francisco, CA 94132, USA; jvo@sfsu.edu

**Keywords:** CBPR, breast cancer, training, community, partnership, health disparities, environment

## Abstract

This paper describes the development and feasibility of the Community Based Research Infrastructure to Better Science (CRIBS) training. The goal of this training program was to help new or existing community-academic teams to build strong partnerships and successfully develop together fundable research projects focused on breast cancer environmental causes and disparities. A comprehensive mixed-methods participatory approach was utilized to assess the training. Twenty-two community-academic teams applied for the training program; twelve teams were enrolled. All teams completed the training and subsequently submitted research applications for funding. All components of the training received high ratings and positive qualitative comments. Self-rated competency in all of the learning domains increased during the training. Four (33%) of teams were successful in their first attempt to garner research funding, and six (50%) were eventually successful. The evaluation of CRIBS found it to have successfully achieved all four goals of the training: (1) Twelve new CBPR (community-based participatory research) teams, (2) improved knowledge about CBPR and science, (3) twelve submitted grant proposals in the first year, and (4) six (50%) successfully funded research projects.

## 1. Introduction

Community-based participatory research (CBPR) pairs academically-trained scientific researchers with community members to partner in all steps of the research process [1]. The benefits of conducting CBPR include improved research quality, strengthened communities and researchers, and improved public health [2,3,4]. This paper describes the development, implementation, and process evaluation of a collaborative training program designed to stimulate CBPR addressing the environmental causes of and disparities in breast cancer. The results demonstrate that teams can be taught to develop partnerships capable of designing research projects that are well received by peer reviewers.

The California Breast Cancer Research Program (CBCRP) of the University of California is the largest state-funded breast cancer research funder in the nation. In 2001 a comprehensive review of its and other agencies’ funded portfolios revealed a lack of investigator-initiated research focused on the role of the environment and breast cancer disparities, both areas of programmatic and community interest [5]. To redress this gap, CBCRP sought to focus attention to these issues through one of its major funding mechanisms, the Community Research Collaboration (CRC) award for CBPR projects proposed by community-academic teams. There are two types of award. A pilot CRC 18-month award provides teams with up to $150,000 along with additional funds for indirect costs. A full CRC three-year award provides teams with up to $600,000 plus indirect costs. For both award types, applications are evaluated in a two-tier review process that includes an NIH-approved scientific peer review process and review by CBCRP Council for seven programmatic criteria. In 2000 and 2007, the CRC awards were evaluated and found to be successful in bringing community-academic partnerships together to conduct breast cancer research. These evaluations demonstrated that teams with the most effective working relationships have the most research impact. Areas of difficulty for all teams include collaborative data analysis, power sharing, and managing the impact of turnover [6,7].

In 2010, CBCRP partnered with Commonweal (a nonprofit community-based organization) and Plumbline Coaching and Consulting (a community-based training firm), henceforward the training team, to develop and pilot-test an intensive training program—Community-based Research Infrastructure to Better Science (CRIBS) to address the areas of difficulty identified in the previous studies. Commonweal is a nonprofit cancer retreat center and a convener of thought leaders for scientists and advocates to learn about and contribute to protecting human health and a healthy environment. Plumbline creates and conducts training programs in CBPR and public policy advocacy. The goal of the CRIBS training was to help new and existing community-academic teams build partnerships and develop fundable research projects focused on breast cancer environmental causes and disparities. Subsequent to completion of the training program, CBCRP anticipated the submission of a competitive CRC grant application from training program participants.

## 2. Methods

The training team built a community-academic CBPR partnership, who met over twelve months to develop the outreach strategy and curriculum, select fellows, and conduct the training. Throughout the process, each party carried out specific tasks, and each party had equal decision-making authority. The training team took turns hosting meetings and, through collaboratively created agendas and meeting facilitation, recorded tasks each partner undertook to move the project forward. Team members presented their work at each meeting, allowing the training team to learn from each other and together develop the curriculum and individual sessions. Decisions were made by consensus. The project was reviewed and approved by the Institutional Review Board at the University of California, Berkeley.

### 2.1. Outreach

Candidates were recruited through eleven outreach workshops in areas across the state of California, at both community and academic venues. To choose workshop locations, the training team took a map of California and with shared knowledge of roads and mountains, determined eleven locations that the majority of Californians could reach. In total, 272 people attended these outreach workshops. In addition, the training team engaged in extensive electronic outreach to academically-trained researchers and a wide variety of breast cancer, environmental and women’s health organizations. Teams consisting of at least one academic partner and one community partner were eligible to apply. Applicants were self-identified pairs of one academic and one community partner who were in a new or existing partnership, interested in using CBPR to explore breast cancer disparities or environmental contributors to breast cancer. Teams were required to identify partners on their own and to apply as a team, but were not required to have experience working together. Some had met at the outreach meetings described above. Each team submitted a joint application that included their individual and joint community and research experience, and their initial research goals. Twenty-two community-academic teams from throughout the state of California submitted applications; applications were reviewed by all members of the training team using defined criteria and a standard review form, followed by a review meeting, during which the training team reached consensus on the acceptance of 12 teams, comprising 33 individuals (19 community partners and 14 academic partners). Of the 32 participants providing demographic data, ten (56%) of the community partners and seven (50%) of the academic partners were ethnic minorities. The mean age for both the community partners and the academic partners was 50.

Academic partners who had not previously obtained an independent investigator grant (National Institutes of Health Research Project Grant (R01) or equivalent) were considered for the training if they could demonstrate that a mentor would work with them to ensure they had the necessary support of a successful grant writer. Seven (58%) of the teams included academic partners who required and obtained a mentor.

### 2.2. Training Objectives

The training team first conducted an assessment of previously developed CBPR training materials, written information, and peer-reviewed literature. Twenty-five trainings, in addition to numerous other collaboration documents, were identified through web-based searches and a request for curricula sent to the Community Campus Partnerships for Health (CCPH) listserv. A copy of the annotated CBPR training curricula reviewed is available from the authors.

Taking into consideration the design elements of the reviewed CBPR training programs, the CRIBS training team sought to fill a significant gap. Namely, the team developed a CBPR training program where community-academic teams could receive a significant amount of face-to-face, telephone, online, and webinar training and technical assistance. The training was designed so that teams would build skills and create a CBPR project described in a competitive grant proposal in the area of disparities in breast cancer and/or environmental causes of breast cancer.

Further, given prior research findings that lack of scientific knowledge can limit community members’ ability to act as full partners, the training was structured to include education on scientific content around breast cancer, disparities, and the environment [8]. The training structure, including academic and community partners together for all sessions, was also intended to create stronger partnerships. Equal attention was paid to curriculum content that addressed previously noted areas of collaborative difficulty (e.g., collaborative data analysis, power sharing, and managing the impact of turnover). Content was built into the curriculum that would support teams in articulating and documenting their plans for collaborative data analysis. Further, experiential assignments required that teams create a collaborative agreement. These agreements delineated specific procedures for handling such areas as data ownership and sharing, disagreements, plans for broader community involvement in all phases of the research project, plans for dissemination of results, as well as conscious decision making about and a process for expanding the team to ensure a broad enough team to fill vacancies. Finally, specific partnership and scientific mentoring were created for each team. The complete training program had four objectives described below.

#### 2.2.1. Training Objective 1: Creating and Maintaining CBPR Teams

Several theoretical and conceptual models for strengthening the characteristics of CBPR partnerships helped inform the curriculum [9,10,11,12]. The curriculum team determined that the following elements related to community and academic partnership should be addressed in the training:

- Individual motivation and personal goals to help the partners learn about each other and find purpose and goals for the partnership;
- Ability to work well with each other including understanding the differences and similarities of academic and community-based institutional pressures;
- Creating institutionalization and sustainability of CBPR within academic and community institutions;
- Dealing with conflict, while maintaining a strong relationship;
- Creating partnership agreements and team evaluation methods and timeline;
- Expanding the team to include additional team members;
- Establishing team agreements, including decision making and how to handle conflict;
- Dissemination to both community and academic audiences.

#### 2.2.2. Training Objective 2: Understanding the Science of Breast Cancer Prevention

The training team determined that information and training related to the environment and disparities in breast cancer should be included in CRIBS for both the academic and community partners. This included neighborhood and social factors, such as racism and its associated stress, the physical and chemical exposures where people live, work, and play, as well as the lifetime exposure to chemicals and windows of susceptibility, and the ways these factors can be addressed to reduce incidence and mortality of breast cancer.

#### 2.2.3. Training Objective 3: Creating a Pathway from Idea to Funded Research Project

In order for a research idea to move progressively from vision to detailed project stage, a number of factors must be addressed, including developing specific aims and detailed methodology. To ensure that both academic and community partners could fully participate in developing the research plan, training was included on scientific methods, how to conduct literature reviews, statistical concepts, scientific certainty, and understanding how to break up bigger research questions into more feasible research project questions.

#### 2.2.4. Training Objective 4: Writing Successful Grant Applications

Writing research proposals that will go through a rigorous scientific peer-review is generally unfamiliar to most community groups. Understanding the key research grant proposal sections, how to prepare an equitable CBPR budget, and how the grant review process is conducted was shared to provide CBPR teams with mutual knowledge of their path forward.

### 2.3. Training Program

**Figure 1:**
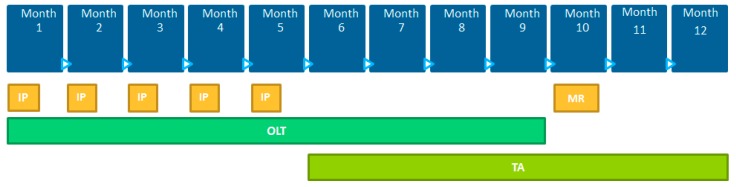
Structure and timing of Community Based Research Infrastructure to Better Science (CRIBS) training program. Notes: IP = in-person meetings; MR = in-person mock review of applications; OLT = online training and webinars; TA = telephone-based technical assistance.

The structure of this training was based on best practices of the adult learning literature: Highly interactive, drawing on high personal motivation, and accounting for different learning styles. There were three primary formats of training provided: Face-to-face, online and telephone-based. Figure 1 provides an overview of the timing and types of training offered during 12 months of CRIBS.

Table S1 provides the details of the course objectives, sessions, and training methods. The three types of training methods are described here: 

(1) Face-to-Face (FTF): Five two-day FTF trainings were delivered in months 1–5 with sessions that corresponded to the four stated objectives of the program.

The FTF sessions were each two days long and held in a consistent California location (CRIBS covering hotel and travel expenses). These allowed for ample time for training sessions and team work sessions, as well as team and cohort downtime to continue relationship building. Sessions conducted by academic and community trainers covered CBPR and partnership building, health disparities in and environmental causes of breast cancer, the research process and grant writing, as well as dissemination techniques.

(2) Online Training (OLT): Ten OLT sessions.

Ten OLT sessions and three webinars were delivered in months one through ten. Specifically, OLT sessions using a University-based online teaching platform and online webinars took place in between and after the FTF trainings. The online format encouraged active participation over the 12-month training period.

(3) Telephone-based: Three technical assistance (TA) telephone calls of 50 min for each CRIBS team were delivered to support the team’s integration of knowledge of CBPR, health disparities, the environment, and the research process.

Trainers provided written and verbal feedback on 8-page concept papers written by participants. An in-person “mock” grant review was held in which draft CRC proposals from each team were reviewed by former CRC reviewers with fellow teams present and observing.

### 2.4. Evaluation

An outside evaluator developed a mixed-methods evaluation framework utilizing a participatory evaluation approach. Activities included an online demographics questionnaire; online pre-training and post-training self-rating of research and partnership competencies; online anonymous satisfaction and feedback evaluations after each FTF training and throughout the TA period; and a follow-up survey after submission of the final CRC grant proposal, but prior to the announcement of funding results. Response rates across online surveys were between 91% and 100%.

#### 2.4.1. Demographics Questionnaire

The 80-item demographic questionnaire was comprehensive and included skip patterns to reduce participant burden. Participants responded to questions on areas such as ethnicity, age, gender, sexuality, income, and education. Several content areas included additional questions, such as nationality and immigration, neighborhood characteristics and resources, number and type of people in the household and contributing to income.

#### 2.4.2. Pre-Training and Post-Training Questionnaire

Each assessment consisted of 62 questions examining current competence in six learning domains (scientific research; CBPR; partnership; funding; disseminating results; breast cancer science). Competency was self-reported on a scale from 1 (lowest) to 5 (highest). Mean values and standard deviations were computed for each of the learning domains in both pre-training and post-training competency. Due to the small sample size (*n* = 31 providing data at both time points) a Wilcoxon signed-rank test was used to analyze the data, examining the change in each participant’s competency from prior to the training to after the training. STATA 13 (StataCorp LLC, College Station, TX, USA) was used to conduct statistical analyses; significance was assessed at *p* < 0.05.

#### 2.4.3. Satisfaction and Feedback Survey

The satisfaction and feedback surveys that covered the related online trainings, webinars and in-person sessions were completed regularly throughout the CRIBS program, following FTF sessions. Depending on the number of sessions and faculty, the surveys ranged from 14–37 questions. Using a scale of 1 (poor) to 5 (excellent) participants rated the quality of each session and associated faculty. Participants rated whether they had learned the primary objective of each session by using a scale of 1 (strongly disagree) to 5 (strongly agree). Several open-ended questions were included for participants to provide more detailed feedback. Unique identifiers were used to make sure that duplicate responses could be identified. Identifiers were a combination of participant’s birth month, birth year and first name of participant’s mother. Mean values and ranges of scores were computed for each of the quantitative questions in Microsoft Excel, and no statistical comparisons were made across ratings of sessions or presenters.

#### 2.4.4. Follow-Up Questionnaire

A follow-up questionnaire was completed in 2013 between the spring submission of applications and summer notification of funding results. Participants answered questions on aspects of designing, preparing and submitting their Community Research Collaboration applications, and plans to submit additional applications. Response options ranged from 1 to 5 for quantitative questions, and question anchors depended on question content (for example, Strongly Disagree to Strongly Agree, or Not at All Involved to Very Involved). Several open-ended questions allowed participants to provide feedback on how they might have improved their team process.

Qualitative telephone interviews were conducted to collect additional satisfaction and feedback data. In this paper we have focused on reporting results from quantitative and qualitative data collected via online questionnaires and surveys. Presentation of the full set of telephone interview results is beyond the scope of this paper.

## 3. Results

The evaluation results are arranged into five areas: Demographics of CRIBS participants, the training’s feasibility, acceptability, and impact (on both the partnership, as well as the quality of the grant proposal), and sustainability.

### 3.1. Demographics of Participants

The CRIBS cohort included 49% ethnic minority participants, the mean age of the group was 51 years, and a majority of the cohort was women. Forty percent of participants had earned a bachelor’s or master’s degree, and 37% had completed a PhD. More than half of the cohort was community partners. See Table 1 for details of demographic characteristics.

### 3.2. Feasibility of Training

Participation in this time-intensive training was high. Of the 33 participants providing data, 21 (64%) participants attended all ten FTF training days and the mock review. Of the twelve (36%) who missed any part of the 10 days or mock review, six (18%) missed no more than 2 days and one (3%) person missed five days, due to a family death. One (3%) person formally withdrew from the training program and one (3%) additional person discontinued their participation during the training program without formally withdrawing.

OLT participation was more uneven. For ten OLT sessions (which contained a total of 12 segments, as two of the sessions had two distinct segments), participants were asked to post at least once for each segment and respond to at least seven other posts at some point during the program, for a total expected post/response rate of 19. The average post/response rate was 21.4, median 20, but ranged from 8 to 49.

Evaluation participation was strong. The online surveys were completed by a majority of participants, with 32 (97%) responding to the demographic questionnaire, 31 (94%) completing the pre-post questionnaires, between 27 (82%) to 31 (94%) completing five satisfaction and feedback surveys and 28 (85%) completing the follow-up survey.

### 3.3. Acceptability of Training

Participants rated all aspects of CRIBS highly (see Table 2). In all but one close-ended question, the majority of participants endorsed the sessions and the faculty as either “very good” or “excellent.” On a scale of 1 (low) to 5 (high), the Mock Study Section Review scored an average of 4.86 with FTF sessions averaging 4.46. Participants favorably evaluated the CRIBS faculty (both core trainers, as well as guest faculty), and the open-ended questions revealed enthusiasm for the topics covered, the structure of the presentations, and the specific speakers. The average score for FTF presenters was 4.40, and 3.86 for webinar presenters.

The main findings from open-ended comments related to each of the training components revealed:

#### 3.2.1. FTF Component

- Participants felt that the curriculum (presentations and activities) was exemplary;
- Teams benefited greatly from the partnership building activities;
- The environment supported co-learning among participants and between participants and trainers/speakers;
- Participants appreciated receiving detailed feedback from the Mock Review;
- The opportunity to observe the Mock Review benefited participants.

#### 3.2.2. OLT Component

- Benefits of OLT included access to valuable resources, learning opportunities, and interaction with other participants;
- Challenges of OLT included time constraints and the difficulty some participants had engaging in the online environment because they did not feel stimulated, did not feel they had much to contribute, and/or were not comfortable communicating with others in an online forum;
- Webinars were positively received although technical problems with one webinar compromised its quality.

#### 3.2.3. Telephone TA Component

- Mentoring and feedback were very helpful to the teams;
- Some teams were frustrated and/or confused when they received conflicting feedback from the CRIBS trainers and mock reviewers;
- The TA, particularly the mock review debrief, helped validate feelings and concerns;
- The TA was helpful in answering team’s specific questions.

### 3.4. Impact of Training

Comparisons of pre-training and post-training self-rated competencies indicate that CRIBS participants improved their competency in all six learning domains (Table 3). Out of the six learning domains mean competency scores increased by 13% to 46%.

When stratifying by type of partner (community or academic), the three greatest improvements for community partners were for CBPR (78.8% improvement), breast cancer science (64.5%) and partnership knowledge and skills (44.28%). Community Partners also showed significant improvements in scientific research (22.36%), funding (29.62%), and disseminating results (25.9%). For academic partners, due to the high self-rated competencies in the pre-training assessment, significant improvements were found in only two areas: Breast cancer science (25.14%) and disseminating results (9.7%).

CRIBS participants overwhelmingly felt the training strengthened their knowledge and understanding of breast cancer science and CBPR, their grantsmanship skills, and their partnerships. Of particular note, among the 28 participants providing follow-up survey responses, both community and academic partners reported that their research plans included a strong focus on collaborative analysis. On a scale of 1 (Low) to 5 (High), both community participants and academic participants reported strong agreement that their research plan provides community partners with significant opportunities to participate in the planning and executing of data collection (4.77 community; 4.90 academic), the planning and/or executing analysis (4.76 community; 4.91 academic), and the plan involves the community partners in interpreting the research findings (4.88 community; 4.82 academic).

When articulating their role in each stage of the preparation and submission process for their CRC application, community and academic participants indicated equity and power-sharing in their responses. Both community and academic participants noted that were very involved in defining the problem on which to focus (4.94 community; 5.00 academic), developing their research question(s) and specific aims (4.94 community; 5.00 academic), developing their collaborative agreements (4.83 community; 4.73 academic), developing their budget (4.53 community; 4.45 academic), developing their research plan (4.59 community and 5.00 academic), and submitting their application (4.59 community; 4.55 academic).

Participants shared many positive comments about the training program, partnership building opportunities, and their ability to learn from each other. Table 4 presents sample comments of satisfaction shared by participants:

During the 12-month training period, ten (83%) teams stayed together and two (17%) teams reconfigured (one due to interpersonal conflict, and the other due to a mismatch between the community’s topic of interest and the academic partner’s expertise). The two teams that chose on their own not to continue working together were supported by the training team in identifying new partners who were brought into the training and provided access to previous session materials, videos and webcasts.

Following the 12-month training period, the academic partner in one team secured a new position, and the team was able to engage in a real-world test of managing the impact of turnover, (an area of difficulty reported by CRC teams in the past). The team openly communicated about the change among themselves and with CRIBS staff and engaged in a collaborative process of identifying a potential new partner, discussing the collaborative fit and ultimately forming a team with a senior colleague of the original academic partner. The team has maintained their partnership for seven years and has been funded a total of four times by CBCRP.

All twelve CRIBS teams submitted pilot applications to CBCRP that were competitively peer-reviewed together with applications from non-CRIBS applicants through their usual review process [13]. The review committee was composed of academic and community advocate reviewers who were not made aware of the CRIBS training or which applicants participated in CRIBS until after the review was completed. The applications were then subjected to the second step of the standard review process, programmatic review by members of the California Breast Cancer Research Council, who were also blinded to the CRIBS status of applications. Teams were permitted to discuss their participation in CRIBS in their application as a way to document how they developed their partnership, but CBCRP did not inform the reviewers to avoid biasing the reviews.

Of the 12 applications from CRIBS fellows, four (33%) were successful in their first attempt to garner research funding and six (50%) were eventually successful. More specifically, four of the twelve applications submitted in 2013 were funded; one additional application was funded via resubmission in 2014; and in 2019, one more team was funded. In total, six out of 12 CRIBS teams (50%) were funded through CBCRP as of 2019. Further, four of the five (80%) of CRC pilot grant applications funded in 2013 by CBCRP were from the CRIBS cohort.

### 3.5. Sustainability

While formal data on sustainability were not part of the evaluation plan for the program, the training team tracked the number of teams completing the various components of the training, as well as the number of teams submitting applications between 2013 and 2019. Ten teams stayed together with original partners throughout the training program and mock review and two teams reconfigured (one due to interpersonal conflict, and the other due to a mismatch between the community’s topic of interest and the academic partner’s expertise). Of the six teams that were funded between 2013 and 2019, three have sustained their partnerships for at least four years, with two of these staying together for at least seven years. Of the two seven-year partnerships, one secured CBCRP funding an additional three times between 2014 and 2019 and the second team an additional two times. Of the six teams that were not funded between 2013 and 2019, four sustained their partnership for at least two years and submitted applications to CBCRP in 2014 (three teams) and 2016 (one team). We have no knowledge of any teams receiving funding from other agencies.

The training team developed a deep respect for each other through this work and has continued to work together, obtaining NIH funding for adapting this training into a short course.

## 4. Discussion

Community-based participatory research (CBPR) is by definition a collaborative process. While the theory and practice of CBPR can be and often is taught to individuals, this does not allow for experiential learning, one of the most powerful adult learning methods available [14,15,16,17,18,19]. For this reason, we and others who have developed CBPR training programs believe it is important to train teams, not just individuals, in CBPR [20,21,22,23]. Given our own and others’ findings on the impact of the degree of collaboration, almost all existing trainings provide both didactic and experiential training on partnership and collaboration [20,21,22,23,24,25,26,27,28,29,30,31,32,33,34,35,36,37,38,39]. Based on this study, we found that team members were able to use the training time to build or reconfigure their partnership. In addition, we concur with the findings of Andrews et al. that “training in a group format allowed the partners to learn from other CBPR teams, having multiple community partners in the same training provided more empowerment for community voice to “negotiate transactions and assume equitable power [21]”. Teams become role models, examples, and resources for each other as they foster greater collaboration and shared ownership of their research projects, building capacity among academic and community researchers alike.

The CRIBS Training introduces some unique and important elements. Due to the previously-documented knowledge gap between academic and community researchers in science, and the impact this gap can have on power dynamics, we also included knowledge in science and scientific methods. This was possible because all teams were working in a single disease area. An equalizing factor is that both academic and community researchers learned new areas of science, since the topics covered two very disparate areas (social determinants of health/ health disparities and environmental contributors to breast cancer). Team members heard the same information about scientific methods and breast cancer science, both learning new science and methodology, and were able to discuss and find alignment in their understanding of their research. In addition, as Fording also found, guiding teams to openly discuss each partner’s needs and expectations, as well as factors leading to differential power and authority and ways to address these imbalances, improved power dynamics [40].

Another unique aspect to the training was the use of a personality inventory (Myers-Briggs Type) as a tool to help teams relate to each other as equal team members, each with strengths and challenges [41]. This session allowed partners to communicate about their differences using similar language. This was a uniformly well-rated and popular session that generated intense conversations within teams and was referred to often in later sessions. It was perhaps the tipping point to authentic personal relationships between partners.

An interesting finding was the differential pre-training self-assessment of competencies between academic and community researchers. While it is not surprising that academics rated themselves highly on scientific research and breast cancer science, it is striking that they also rated themselves more highly on CBPR, partnership, funding and dissemination as these are factors that are critical to success in community work, as well as academic research. We found no other trainings that measured pre and post-test competencies except one and those self-assessments were not reported separately for academic and community researchers [20]. The training team’s sense is that the academic researchers improved more in the competencies than reflected in their self-assessment, suggesting that a more objective measure might have been used. This differential pre-training self-assessment of competencies between academic and community researchers could contribute to the power differential often witnessed in community-engaged research, and it warrants further exploration in future studies.

Another indicator of a successful training program is the 33% funding rate for first time grant proposals, and 50% overall funding rate. Both are high funding rates compared to CBCRP and many other funders, including National Cancer Institute, which reported success rates of 11.3–14.0 per cent in 2013–2018 [42].

To the best of our knowledge, CRIBS remains unique in several ways:

1. It was designed to increase the number of CBPR teams conducting research on two understudied areas of breast cancer—environmental causes of breast cancer and disparities in breast cancer;
2. It trained both new and existing CBPR teams to build a collaborative CBPR partnership;
3. It offered participants a mock grant application review, providing an insider perspective on the decision-making process seldom experienced by research grant applicants;
4. It coordinated the timing of the training so that CBPR teams were fed directly into a relevant funding cycle.

## 5. Conclusions

Overall, CRIBS was a successful model for building capacity for community-academic partnerships to conduct CBPR. While there are opportunities for improvement, the overall approach holds promise as a model for supporting more teams to successfully build CBPR partnerships and develop scientifically rigorous research projects that are meaningful to communities. Some of the key lessons that can be applied to future trainings include the following:

- Training is effective: Intensive training in CBPR can successfully build new and strengthen existing partnerships, as well as increase the chance that teams will be successful in receiving research funding.
- Team training has benefits: There are considerable benefits to training the academic partner and community partner together. Time together in a learning environment allowed teams to build agreements on how to work, develop a common language around the issues they want to address and test their compatibility in skills and working styles before beginning a multi-year working relationship.
- Teaching science and partnership skills deepened the experience: Combining training in both science and building a CBPR partnership allowed teams to gain a more holistic and grounded approach to the entire spectrum of responsibilities associated with conducting CBPR.
- Mock reviews added value: The mock review provided a realistic perspective on the strengths and weaknesses of the teams’ proposal, as well as insight into the wide range of interpretation reviewers may have to a proposal. We know of no other trainings that provide such an opportunity for grant applicants to experience such a realistic perspective on their work.
- Having a funding source matters: Connecting training to an opportunity to apply to a specific funding source strengthened the training. Specifically, it allowed experiential training sessions to be focused on results (for example, training on how to develop a CBPR budget that connects directly to the budget requirements of CBCRP’s CRC grant application). Additionally, it gave teams a goal and a deadline to work through their process.

Some of the areas for improvement learned from the CRIBS training include:

- Shorter training: Many participants felt the training was valuable, but possibly too time intensive.
- More interaction: Teams expressed a preference for more unstructured time with their partners to develop research ideas and build their partnership.
- Tailor sessions to the participants’ needs: All sessions were required for both academic and community partners. Future trainings could split the partners for some sessions.
- Discuss the challenge of conflicting feedback more extensively: Even though this was raised, teams were still frustrated by conflicting feedback. More emphasis on the inevitability of conflicting feedback and how to work with it could be helpful.
- Improve the quality of webinars: Use a better platform to ensure that trainers and participants can interact smoothly.
- Proactively intervene when teams are challenged: As mentioned, two teams were reconfigured between the beginning of the training and submission of the grant proposals. CRIBS trainers should intervene at the earliest signs of trouble for teams, to assist their working through issues or restructuring.

Developing and implementing CRIBS provided a significant opportunity to build capacity in California for CBPR teams to conduct research on the environmental and health disparities links to breast cancer. The high success rate of CRIBS teams competing for research funding indicates that the extensive effort of the training paid off. The CRIBS model has already served as a template for a condensed program by the same training team. As future trainings are offered, new adaptations will be made with the goal of building a more robust pool of academics and community members in California who are capable and experienced in CBPR.

## Figures and Tables

**Table 1:** Demographic characteristics of CRIBS participants (*N* = 32).

Demographic Characteristic	All (*n* = 32)	Community (*n* = 18)	Academic (*n* = 14)
Community or Academic Partner	-	54%	46%
Age (mean)	51	51	50
Female	83%	84%	81%
Lesbian or Gay	3%	-	6%
Born outside of US	29%	16%	43%
Race/Ethnicity			
Latino	17%	26%	12%
American Indian/Alaskan Native	6%	11%	-
Asian	17%	11%	29%
Black/African American	14%	21%	6%
White	51%	47%	52%
Multi-Ethnic	11%	11%	13%
Household Income			
≤$24K	3%	5%	-
$25K to $75K	31%	37%	25%
≥$76K	65%	58%	75%
Language Other than English Spoken at Home	17%	11%	25%
Highest Education			
GED	3%	5%	-
Some College	9%	16%	-
B.A./B.S.	14%	26%	-
M.A./M.S.	26%	42%	6%
Prof degree	11%	5%	19%
PhD	37%	5%	75%

**Table 2:** CRIBS training sessions and trainer ratings.

**Type of Session**	**# of Sessions**	**Ave Score**	**Lowest Session Score**	**Highest Session Score**
Face-to-Face	31	4.46	4.06	4.88
Webinars	2	4.12	3.94	4.30
OLT	7	4.21	3.89	4.52
Mock Study Section	1	4.86	4.86	4.86
Phone TA	3	4.37	4.25	4.53
**Trainers**	**# Trainers**	**Ave Score**	**Low Score**	**High Score**
Face-to-Face Presenters	53	4.40	3.28	4.92
Webinar Presenters	5	3.86	3.31	4.43
Phone TA Leader	16	4.38	3.25	5.00
In-person TA Leader	13	4.39	3.14	4.86

**Table 3:** CRIBS pre- and post-training self-rated competencies (*n* = 31).

**Total Group** **(*n* = 31)**	**CRIBS Learning Domain**	**Pre-Training**	**Post-Training**	**Difference**	**Percent Improvement**	***z* Value**	***p* Value**
	Scientific Research	3.34	3.78	0.435	13.03%	3.167	0.0015 **
	CBPR	2.49	3.57	1.08	43.3%	3.509	0.0004 ***
	Partnership	2.71	3.435	0.73	26.9%	2.803	0.0051 *
	Funding	3.21	3.78	0.57	17.7%	2.911	0.0036 **
	Disseminating Results	2.89	3.41	0.52	18.03%	3.717	0.0002 ***
	Breast Cancer Science	2.56	3.75	1.19	46.3%	4.140	0.00005 ***
**Community Partner** **(*n* = 18)**	**CRIBS Learning Domain**	**Pre-Training**	**Post-Training**	**Difference**	**Percent Improvement**	***z* Value**	***p* Value**
	Scientific Research	2.826	3.458	0.6319	22.36%	2.573	0.0101 *
	CBPR	2.135	3.818	1.6825	78.8%	3.267	0.0011 **
	Partnership	2.55	3.681	1.1296	44.28%	2.919	0.0035 **
	Funding	2.938	3.808	0.87	29.62%	2.679	0.0074 *
	Disseminating Results	2.549	3.209	0.6605	25.9%	2.919	0.0035 **
	Breast Cancer Science	2.378	3.911	1.53	64.5%	3.575	0.0004 ***
**Scientific Partner** **(*n* = 13)**	**CRIBS Learning Domain**	**Pre-Training**	**Post-Training**	**Difference**	**Percent Improvement**	***z* Value**	***p* Value**
	Scientific Research	4.057	4.22	0.16	4.03%	1.604	0.1087
	CBPR	2.989	3.23	0.24	8.09%	1.192	0.2334
	Partnership	2.923	3.096	0.17	5.9%	0.210	0.8337
	Funding	3.58	3.7350	0.15	4.3%	0.699	0.4844
	Disseminating Results	3.358	3.68	0.325	9.7%	2.315	0.0206 *
	Breast Cancer Science	2.815	3.52	0.708	25.14%	1.961	0.0499 *

* *p* < 0.05, ** *p* < 0.005, *** *p* < 0.0005.

**Table 4:** Illustration of CRIBS fellows’ perspectives and voices.

Theme	Quotes
**Training Program**	Those (CBPR projects presented) were really good examples of how the community responds to research and how we can go about doing work with them.
	I think it doesn’t matter what our economic situation is—I think our life experiences are very, very similar. And I think economics makes it harder or softer, but we have these emotional threads that go through us as black women—a lot of us can relate to a lot of the same things. And so what she found [trainer Sarah Gehlert], when she put science to what is so much in our heart and in our head, that impacted me personally.
	... another thing I thought was really good was seeing the team of people who coordinated the training in action, because a lot of the stuff they were teaching us, they were actually role modeling for us as well … I got to see how they broke up roles and responsibilities and how they kind of worked out all the logistics. And so that was really helpful.
**Partnership Building**	Face to face trainings were the most beneficial for several reasons. It was one of the times where the entire team was able to meet, spend time together, and get to know each other as well as other CRIBS teams. It also provided opportunities for team members to hash out ideas and have their work critiqued (which took place during the training activities). The face to face trainings also served as an impetus to get assignments done and be accountable to each other. The major advantage of face to face trainings is that it takes team members away from the other commitments/responsibilities which might otherwise serve as a barrier to making progress on the team project.
	Being together with our partners was really a great way to get to know one another also, just spending that time with one another.
	… the Myers-Briggs exercise was really great for kind of solidifying things with using terms of appreciating our personalities and finding ways to adapt, you know, and to minimize misunderstandings and … take into account each person’s personality traits …
**Learning from each other**	I also appreciated not only hearing from the experts but hearing from others in the audience about environmental risks in their particular geographic areas and becoming aware that some of the concerns are statewide but some concerns are very specific to geographic areas.
	So, I enjoyed hearing from other partnerships (about) some of the challenges they might be facing or the ways they’ve been successful. And also the ideas they’re generating as well as the opportunity to network for the potential for broader partnerships across the state. So, I made several connections and people that I have linked outside of our county. Outside of the county where we’re partnered and outside the county where I work because we came together with all the partnerships.
	… it definitely had the feeling that we were a group that was being formed. Yes, we had our own individual teams, but also as a group we were in formation, and we were able to form some bonds. And, just the whole creative process is better when you have a group dynamic.

## Supplementary Materials

The following are available online at https://www.mdpi.com/1660-4601/16/22/4310/s1, Table S1: CRIBS COURSE OBJECTIVES, SESSION, AND TRAINING METHODS.
