# Supplementary material for: Development and Evaluation of a Training Program for Community-Based Participatory Research in Breast Cancer
Source: Int J Environ Res Public Health. 2019 Nov 6;16(22):4310. doi: 10.3390/ijerph16224310 (PMC6888545; doi:10.3390/ijerph16224310)
Supplement: Supplementary file 1 [file ijerph-16-04310-s001.pdf]

International Journal of
Environmental Research and Public Health

MDPI

Supplemental Material

This table S1 shows the sessions developed to address each objective, the format used, and what training method was used.

Table S1. CRIBS COURSE OBJECTIVES, SESSION, AND TRAINING METHODS.

Training Objective	Topic	Learning Outcome	Format	Training Method
Training Objective 1. Creating and maintaining community-based participatory research (CBPR) teams.	Overview of CBPR	Learn about and discuss CBPR	FTF	Presentation & Discussion
	Case Study: CBPR as Relevant, Rigorous Research	Hear from a CBPR team about their experience conducting research		Presentation & Discussion
	Vision	Create their team vision		Individual and Small Group Activity
	CBPR Benefits and Challenges	Understand the benefits and challenges of CBPR for both the community and academic partners and will be able to identify benefits and challenges of participating in CBPR for their own partnership	OLT	Video & Discussion
	Seeing Differently	See how individual experiences shape perspective and perception of events	FTF	Interactive group exercise
	Partnership Agreements	Create a draft of their partnership agreements and share it with others.	OLT	Team Posting & Discussion
	Institutionalization and Sustainability of CBPR	Report on their goal(s) for increasing the use of CBPR in their organization, institution, or community, and the resources they need or have to support their effort.		Fellows Posting & Discussion
	Institutionalization of CBPR	Learn how to ensure their institution/organization is supportive of CBPR and institutionalizes the method.	FTF	Small Group Activity
	Partnership Assessment	Learn to Create a self-assessment tool, conduct a self-assessment, and debrief the results.		Team Activity & Discussion
Training Objective 2. Understanding the science of the environmental causes of and disparities in breast cancer.	Risk Factors for Breast Cancer: What We Know and What We Don't Know	Learn about the general risk factors of breast cancer and the complexity of risk factors	FTF	Presentation & Discussion
	New Paradigm of Breast Cancer Causation	Learn about the "new paradigm" of Breast Cancer causation and prevention		
	Pathways to Breast Cancer	Understand inherited genetics; genetic damage; epigenetics; susceptibility to developmental disruption of hormones; endocrine disruptors		
	Role of Services and Screening in Health Disparities	Learn about the role of services and screening in answering questions about the disparities in breast cancer.		
	Film Screening: *Unnatural Causes*	Learn how population health is shaped by the social and economic conditions in which we are born, live and work.		Video & Discussion
	Appreciating Differences	Learn their Myers-Briggs type and identify work and communication differences within their partnership		Presentation, small group and team work
	Moving From Partners to Teams and How to Handle It!	Learn about the theories of how groups form, including through conflict.		Presentation & Discussion
	Team Agreements	Teams will begin developing team agreements.		Team Activity
	The Role of Social Factors in Breast Cancer	Understand how stressors impact biology, including conceptualization of racism and segregation as chronic stressors.		Presentation & Discussion
	Film Screening and Discussion: *Living Downstream*	Learn about environmental health activism and the value of data.		Video & Discussion
	Case Studies: CHAMACOS Study	Learn about the CHAMMACOS study (pesticides in farm worker community) as well as the experience and lessons Learned in partnering with community members		Presentation & Discussion
	Bus Tour	Have an opportunity to tour various environmental and disparities sites in Oakland.		Large Group Activity
	Research on Discrimination and Health	Learn about the impact of discrimination on health and suspected links to cancer.		Presentation & Discussion
	Breast Cancer Disparities and the Environment	Learn what is known about breast cancer disparities and the environment.		
Training Objective 3.	Research Ethics	Understand their obligations to protect the rights and	OLT	Video &

Creating a pathway from idea to funded research project.		welfare of subjects in research and how the ethical principles relate to CBPR.		Discussion
	California Cancer Registry and other Important Data Sources	Learn about different data sources that are available to them.	FTF	Presentation & Discussion
	Scientific Methods Overview	Learn the various scienctic methods that can be used to answer different Research Questions.		
	Research Questions and Methods	Create research questions and possible research designs for those research questions.		Large Group Activity
	Impact of Scientific Methods on Community	Engage with research methods in considering research ethics.		
	Research Questions	Teams will work on their Research Questions.	OLT	Team Posting & Discussion
	Team Work & Staff TA Round Robin	Time for Teams to meet with staff, and on their own, to discuss next steps.	TA	Team Activity
	Method Plan Development	Learn the best practices in how to Create a strong (qualititiave and quantitative) method plan, using theoretical frameworks.	FTF	Presentation & Discussion
	Study Results Dissemination & Impact	Learn about how to have impact & disseminating study results in CBPR.		
	Revised Research Question	Work together to revise their Research Questions based on feedback.	OLT	Team Posting & Discussion
	Team Work & Staff TA Round Robin	Time for Teams to meet with staff, and on their own, to discuss next steps.	TA	Team Activity
	Team Work & Staff TA Round Robin	Time for Teams to meet with staff, and on their own, to discuss next steps.	FTF	Team Posting & Discussion
	Preparation for Concept Paper submission	Refine research question and strengthen their conceptual framework.	Phone	Team Activity
	Review of Concept Paper Feedback	Incorporate staff feedback to strengthen research plan.		
	Cancer Mapping Webinar	Particpants will Learn the utility of cancer mapping and how to incorporate the data into their research.	Web	Presentation & Discussion
Training Objective 4. Writing successful grant applications.	Literature Review	Learn about conducting literature reviews.	OLT	Instructions & Discussion
	Components of a Research Plan	Learn about the various components of a research grant proposal and begin working on their Research Plan.	FTF	Presentation & Discussion
	Institutional Review Boards: Academic and Community-Based	Learn how to work with IRB and IRB applications, especially with CBPR projects, and the value of community-based IRB.		
	Grant Writing from the Grant Reviewers Perspective	Understand about the grant review process and the basic skills necessary in grant writing.		
	Technical Assistance Needs	Identify TA needs.	OLT	Team Posting & Discussion
	Funders Panel	Understand the skills needed in identifying grant opportunities.	FTF	Presentation & Discussion
	CBPR Budgets	Learn the various ways to manage budgets for CBPR.		
	Revised Research Questions	Work together to revise their Research Questions based on feedback.	OLT	Team Posting & Discussion
	Review Plans for Grant Proposal Development	Finalize plan for grant proposals.	Phone	Team Activity
	Application Preparation and Grantsmanship Webinar	Learn grantsmanship skills and what is needed to apply for a CBCRP grant.	Web	Presentation & Discussion
	Preparation for Grant Proposal Submission	Finalizing grant proposal submission plans.	Phone	Team Activity
	Mock Review Preparation Webinar	Learn how to prepare for a grant review.	Web	Presentation & Discussion
	Mock Review	Learn how grant reviews are held.	FTF	Team & Large Group Activity
